# Purification and Immobilization of the Recombinant *Brassica oleracea* Chlorophyllase 1 (BoCLH1) on DIAION^®^CR11 as Potential Biocatalyst for the Production of Chlorophyllide and Phytol

**DOI:** 10.3390/molecules20033744

**Published:** 2015-02-24

**Authors:** Yi-Li Chou, Chia-Yun Ko, Long-Fang O. Chen, Chih-Chung Yen, Jei-Fu Shaw

**Affiliations:** 1 Institute of Biotechnology, National Cheng Kung University, Tainan 70101, Taiwan; E-Mail: qbowchou@gmail.com; 2 Institute of Plant and Microbial Biology, Academia Sinica, Taipei 11529, Taiwan; E-Mails: g874222@gate.sinica.edu.tw (C.-Y.K.); ochenlf@gate.sinica.edu.tw (L.-F.O.C.); 3 Department of Biological Science and Technology, I-Shou University, Kaohsiung 82445, Taiwan; 4 Agricultural Biotechnology Center, National Chung Hsing University, Taichung 40227, Taiwan

**Keywords:** enzyme purification, enzyme immobilization, *Brassica oleracea* chlorophyllase 1 (BoCLH1), DIAION^®^CR11

## Abstract

Recombinant *Brassica oleracea* chlorophyllase 1 (BoCLH1) with a protein molecular weight of 38.63 kDa was successfully expressed in *E. coli* and could catalyze chlorophyll (Chl) hydrolysis to chlorophyllide and phytol *in vitro*. In this study, we used DIAION^®^CR11, a highly porous cross-linked polystyrene divinylbenzene-based metal chelator, for purifying and immobilizing the poly (His)-tagged enzyme. The Cu(II) showed the highest protein adsorption (9.2 ± 0.43 mg/g gel) and enzyme activity (46.3 ± 3.14 U/g gel) for the immobilization of the poly (His)-tagged recombinant BoCLH1 compared with other metal chelators. Biochemical analysis of the immobilized enzyme showed higher chlorophyllase activity for Chl a hydrolysis in a weak base environment (pH 8.0), and activity above 70% was in a high-temperature environment, compared with the free enzyme. In addition, compared with free BoCLH1, the enzyme half-life (*t*_1/2_) of the immobilized BoCLH1 increased from 25.42 to 54.35 min (approximately two-fold) at 60 °C. The immobilized enzyme retained a residual activity of approximately 60% after 17 cycles in a repeated-batch operation. Therefore, DIAION^®^CR11Cu(II)-immobilized recombinant BoCLH1 can be repeatedly used to lower the cost and is potentially useful for the industrial production of chlorophyllide and phytol.

## 1. Introduction

Chlorophyll (Chl) is the abundant pigment involved in the sunlight absorption of photosynthesis, and is annually broken down into more than 10 billion tons in nature. Chl biosynthesis and degradation are regulated by plant development, the environment, and stress response. Chlorophyllase (Chlase, EC 3.1.1.14) is the key enzyme in Chl breakdown during leaf senescence, pod maturation, fruit ripening, and pathogen infection [1]. Chlase hydrolyzes Chl to chlorophyllide (Chlide) and phytol and also hydrolyzes pheophytins (Pheins), which have a structure similar to Chl’s [2]. According to the alignment of the amino acid sequence, all Chlase genes have a highly conserved lipase motif (GXSXG) and a Chlase catalytic triad (Ser-His-Asp), from which the serine acts as a nucleophile in the hydrolysis reaction [2]. Recombinant wheat Chlase (TaCLH) was evidenced to show carboxylesterase activity for ester compounds’ hydrolysis [3]. Moreover, previous studies had pointed out that Chlase might function as a dimeric protein in algae and plants [3,4,5]. The conserved serine of the lipase and the Chlase active site have been substituted with alanine and abolished the Chlase activity. In addition, the substitution of an asparagine or histidine from the Chlase active site to an alanine has been shown to either eliminate or severely reduce Chlase activity, respectively [2,6].

Our previous study successfully isolated three *BoCLH* genes from *Brassica oleracea* and expressed recombinant BoCLHs in *Escherichia coli* for biochemical analysis. Though BoCLH3 was considered likely to be a pseudogene, BoCLH1 and BoCLH2 preferred to hydrolyze Phein, then Chl a and Chl b. A comparison of the kinetic parameters of BoCLH1 and BoCLH2 showed a higher *K*_cat_/*K*_m_ value for BoCLH1, indicating that it has a higher catalytic efficiency than BoCLH2 [2]. Chlide derivatives were demonstrated to have physiological functions that are anti-oxidant [7], anti-cancer [8], and anti-virus [9] *in vitro*. Likewise, phytol derivatives have functions that are anti-tuberculosis [10] and that are involved in apoptosis [11] and the inhibition of inflammation [12]. Therefore, Chlide and phytol derivatives have potential as drugs for the biotechnology and pharmaceutical industries.

Enzymes have high specificity, selectivity, and activity characteristics, and can produce specific substances in industrial application [13]. However, enzymes are easily susceptible to inactivation and usually restricted by a lack of long-term operational stability; also, it is difficult to recycle and reuse the enzyme [14]. Therefore, the development of enzyme-immobilization techniques is beneficial for stabilization of enzymes [15]. Enzyme-immobilized enzymes can be stabilized to prevent intermolecular interactions and enzyme subunit dissociation, promote a structural rigidification and reactivation of the enzyme, and generate a favorable environment for enzyme catalysis [16]. The enzyme immobilization methods can be divided into entrapment, encapsulation, cross-linking (carrier-free), and support binding [14,16,17,18]. The support binding can be bound by physical binding, covalent binding, ion exchange, and affinity interactions to enhance immobilized enzyme stability [19]. In addition, a suitable immobilization method may improve enzyme specificity, selectivity, recovery, activity, storage, and operational stability, and may even be coupled to purification [16,17,18,19,20,21]. 

Enzyme adsorption in immobilized metal ion affinity chromatography (IMAC) occurs through the multipoint interaction between an electron donor group, such as histidine, cysteine, or tryptophan, exposed on the native protein surface and immobilized metal ion of IMAC [22]. Moreover, this method has the risk of desorption between the metal ions and native protein; therefore, native proteins need a multi-interaction, and highly activated or heterofunctional supports of IMAC may be necessary to prevent native protein desorption [23]. The multimeric structure of dimeric enzymes is likely to be stabilized via IMAC supports adsorption, since it is able to form a high ionic strength interaction of enzyme-support to prevent enzyme desorption to the supports [13,24]. Another method is using protein engineering to produce recombinant proteins containing poly (His)-tagged to enhance the strength of the adsorption between one chelate and poly (His)-tagged fusion protein, which has higher selectivity for immobilization than the native protein [21,25,26]. The strength of the adsorption will depend on the different metal natures and concentrations, and affinity ranking of different metal were in the following order: Cu(II) > Ni(II) > Zn(II) ≥ Co(II) ions [22]. Highly activated or heterofunctional supports on IMAC have better efficiency in purifying poly (His)-tagged fusion protein [25,27]. Therefore, IMAC immobilization of poly (His)-tagged proteins is a way to have one step immobilization-stabilization and coupling with enzyme purification on an industrial scale [18,24,25]. Although the covalent attachment on supports has higher enzyme stability than IMAC, the latter is a reversible immobilization that can reuse supports, when the immobilized enzyme lost activity [19,24,26]. In addition, the IMAC technique has other advantages, such as higher recovery, higher selectivity, higher reuse of supports, and lower carrier cost, over other immobilization methods [22,24,25,27]. 

**Figure 1:**
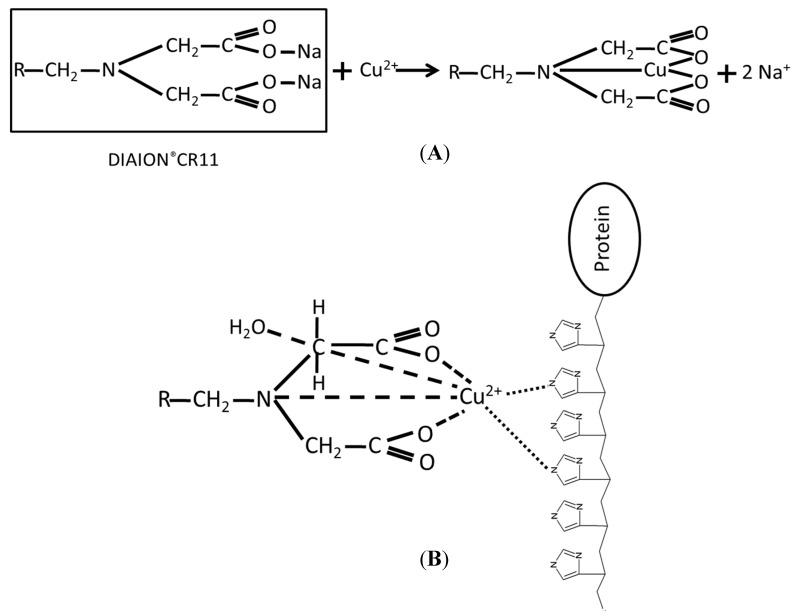
Schematic diagram showing how DIAION^®^CR11 resin with chelated metal ions was used to capture poly (His)-tagged proteins. (**A**) The structure of the DIAION^®^CR11 resin was chelated Cu(II) ions. (**B**) The structure of the DIAION^®^CR11-chelate Cu(II) ions capture the poly (His)-tagged proteins.

One resin of IMAC is DIAION^®^CR11, which is a highly porous cross-linked polystyrene divinylbenzene-based polymer and has an iminodiacetic acid functional group that can be used to capture the chelating metal ions (Figure 1A) [28,29]. At low pH values, DIAION^®^CR11 has extremely high selectivity for chelating divalent metal ions [30]. In Figure 1B, DIAION^®^CR11 chelated metal ions can use specific capture poly (His)-tagged proteins in the crude cell lysate for purifying and immobilizing His-tag fusion proteins. In a previous study, DIAION^®^CR11 chelated with Co(II) ions successfully purified and immobilized His-tag fusion trehalose synthase in a one-step process, and, by using filtration, enabled recycling and reuse for up to 24 cycles [29]. Therefore, immobilizing enzymes with DIAION^®^CR11 resin is a convenient and efficient method for purifying and immobilizing His-tag fusion proteins. This support can be reused by adding an imidazole solution to disrupt interaction between DIAION^®^CR11-chelate metals and enzymes to obtain DIAION^®^CR11-chelate metal for another enzyme immobilization [22,31]. Thus, DIAION^®^CR11 is suitable for reducing costs in industrial production.

In this study, recombinant BoCLH1 was fused with His-tag at the C-terminal and expressed in an *E. coli* system. We used DIAION^®^CR11 to both purify and immobilize the recombinant BoCLH1 in a crude cell lysate and to investigate the biochemical characterization, half-life, and reusability of the immobilized BoCLH1 to compare with free enzyme. 

## 2. Results and Discussion

### 2.1. Immobilization and Purification of the BoCLH1

**Figure 2:**
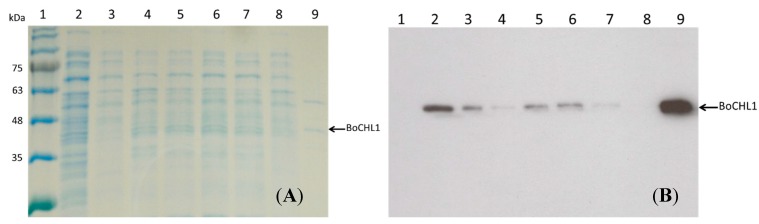
Absorption capacity of metal chelators for metal ions. (**A**) SDS-PAGE analysis of the spent cell lysate after the adsorption of proteins with metal ion-loaded DIAION^®^CR11. (**B**) Western blot was performed using a monoclonal anti-His tag antibody. Lane 1: marker, Lane 2: crude cell lysate, Lane 3: Fe(II), Lane 4: Co(II), Lane 5: Mn(II), Lane 6: Zn(II), Lane 7: Ni(II), Lane 8: Cu(II), Lane 9: purified BoCLH1 (38.63 kDa) was used as a positive control. The arrow indicates the location of recombinant BoCLH1.

To increase the possible industrial applications of the enzyme, we needed to reduce the cost of its purification. Therefore, the immobilized enzyme was a superior choice to reduce cost and increase reused efficiency and stability [32]. For enzyme purification and immobilization, we took six different metal ions of various solutions (40 mL of 200 mM FeCl_3_, Co(NO_3_)_2_, MnCl_2_, ZnSO_4_, NiSO_4_, or CuSO_4_) for chelating reactions with DIAION^®^CR11, along with the crude cell lysate of recombinant BoCLH1. The recombinant BoCLH1 had a C-terminal His tag that could be captured by the metal ion-loaded DIAION^®^CR11 in the crude cell lysate. To confirm the enzyme-binding capacity of DIAION^®^CR11 chelate metal ions and recombinant BoCLH1, the same amount of crude cell lysate was used with each of the six DIAION^®^CR11 metal chelators, and then the unbinding proteins remaining in the spent crude cell lysate after adsorption were analyzed by SDS-PAGE analysis and western blot (Figure 2). 

The binding capacity of DIAION^®^CR11Cu(II) was the best as compared with the other five DIAION^®^CR11 chelate metal ions, whereas the binding capacities of DIAION^®^CR11Fe(II), DIAION^®^CR11Mn(II), and DIAION^®^CR11Zn(II) were poor because the His-tag at the C-terminal of recombinant BoCLH1 showed higher spent crude cell lysate remaining than DIAION^®^CR11Cu(II). This result suggests that Cu(II) can purify and enhance the adsorption of poly (His)-tagged enzymes through the formation of a coordination bond [29]. In addition, the adsorption capacities, immobilization yields, and catalytic activities of the metal-loaded absorbents were further quantified through a protein assay.

### 2.2.Capacity, Activity, and Purification Efficiency of the Adsorbents

We took 1 g dry weight of the immobilized recombinant BoCLH1 mediated by six DIAION^®^CR11 chelated metal ions, eluted them in a 50 mM phosphate buffer containing 500 mM imidazole (pH 7), and calculated the amount of enzyme capacity (mg/g gel) by using a Bradford dye-binding assay. As shown in Table 1, the Cu(II)-loaded adsorbent, 9.2 ± 0.43 mg/g gel, exhibited the maximal protein adsorption, with the next being that of the Fe(II)-loaded adsorbent, 7.9 ± 0.98 mg/g gel, and the minimal protein adsorption being that of the Co(II)-loaded adsorbent, 1.0 ± 0.16 mg/g gel. A previous study reported that the formation of enzyme-coordinated linkage with the chelated Co(II) required the presence of a minimum of two adjacent exposed histidines on a protein surface; however, with the chelated Cu(II), only a single exposed histidine was required for linkage [33]. Therefore, the Cu(II)-loaded adsorbent has a lower selectivity for the poly (His)-tagged proteins than the Co(II)-loaded adsorbent, and the Cu(II)-loaded adsorbent has more protein-adsorption capacity than the Co(II)-loaded adsorbent. This result can correspond to the immobilization curves (Figure S1); the result showed resin chelated with Cu(II) and Fe(II) ions, both of which had a higher protein capacity than the other four ion-chelated metals. Co(II)-chelated resin had the lowest protein capacity for capturing poly (His)-tagged recombinant BoCLH1.

As shown in Table 1, when Chlase was immobilized by a strong interaction with one chelate on supports, the Cu(II)-loaded adsorbent is highest in immobilization yield, 74.3% ± 6.49%, whereas the Co(II)-loaded adsorbent has the lowest immobilization yield, 8.2% ± 1.53%. 

**Table 1:** Characterization of the immobilized BoCLH1 mediated by chelated metal ions.

Chelated Ion	Protein Adsorbed (mg/g gel)	ImmobilizationYield (%)	Enzyme Activity of Immobilized (U *^b^*/g gel)	Specific Activity of Immobilized (U/mg Protein)	Specific Activity of Non-Immobilized (U/mg Protein)
Free BoCLH1	0	ND *^a^*	ND	ND	2.46 ± 0.11
Fe(II)	7.9 ± 0.98	63.7 ± 7.99	12.7 ± 1.36	1.61 ± 0.17	1.80 ± 0.13
Co(II)	1.0 ± 0.16	8.2 ± 1.53	7.3 ± 2.09	7.24 ± 2.07	0.98 ± 0.01
Mn(II)	2.2 ± 0.21	18.1 ± 2.76	1.1 ± 0.2	0.50 ± 0.09	2.03 ± 0.05
Zn(II)	3.1 ± 0.93	24.8 ± 5.92	7.6 ± 1.89	2.5 ± 0.61	1.3 ± 0.04
Ni(II)	4.9 ± 1.76	39.0 ± 10.95	22.1 ± 1.36	4.5 ± 0.28	0.75 ± 0.01
Cu(II)	9.2 ± 0.43	74.3 ± 6.49	46.3 ± 3.14	5.0 ± 0.34	0.39 ± 0.09

*^a^* Not detected. *^b^* The enzyme activity was determined by measurement of the absorbance of the aqueous EtOH phase with Chlide a at 667 nm. One unit of Chlase activity was defined as described in Materials and Methods.

The catalytic activity of recombinant BoCLH1-loaded adsorbents is also shown in Table 1. The enzyme activity of the Cu(II)-loaded adsorbent, 46.3 ± 3.14 U/g gel, was higher than that of other metal ion-loaded adsorbents. The Ni(II)-loaded adsorbent had the second highest enzyme activity (22.1 ± 1.36 U/g gel) and the Fe(II)-loaded adsorbent the third (12.7 ± 1.36 U/g gel), but this result was opposite that of protein adsorption capacity. Therefore, there is no direct correlation between adsorption capacity and enzyme activity; for another example, the protein adsorption of the Co(II)-loaded adsorbent (1.0 ± 0.16 mg/g gel) was less than that of the Mn(II)-loaded adsorbent (2.2 ± 0.21 mg/g gel), but the enzyme activity of the Co(II)-loaded adsorbent (7.3 ± 2.09 U/g gel) was higher than that of the Mn(II)-loaded adsorbent (1.1 ± 0.2 U/g gel). The significant difference in enzyme activity can be attributed to the difference in strength or selectivity of the adsorbents for recombinant poly (His)-tagged BoCLH1. The affinity and specificity are, respectively, described as the strength and selectivity of the cation’s binding capacity, with the higher specificity referring to the cation’s binding with a lesser range of proteins [22,34]; therefore, these two indicators are in opposition to each other. Another reason for the high adsorption capacity may be due to the nonspecific binding between the chelated ions and the surface histidines of enzymes [33]. For instance, DIAION^®^CR11Fe(II) had the second highest protein capacity due to non-specific binding, and its specific activity was poor. On the contrary, DIAION^®^CR11Co(II) had the lowest protein capacity, but higher specific activity than other chelate metals. This result indicated that DIAION^®^CR11Co(II) had the highest specific affinity binding with poly (His)-tagged recombinant enzymes. Moreover, IMAC immobilization marks not only the immobilization-stabilization of an enzyme but also coupling with purification ability [22]. 

The purification efficiency can correspond to the specific activity of metal ion-loaded adsorbents and spent cell lysate. In Table 1 and Figure S1, after Cu(II) immobilization, the supernatant had minimal residual activity (0.39 ± 0.09 U/mg protein) and relativity protein (25.84%) over the other supernatants. This result indicated that DIAION^®^CR11Cu(II) exhibited the best purification efficiency for recombinant poly (His)-tagged BoCLH1. On the contrary, the supernatant of Mn(II) retained 2.03 ± 0.05 U/mg protein specific activity and 81.71% relativity protein, suggesting that the recombinant enzyme still existed in the spent cell lysate. Therefore, the DIAION^®^CR11Mn(II) had the worst purification ability of all the ions. It is interesting that the supernatant of Co(II) retained more recombinant enzyme than supernatant of Cu(II), but the Co(II)-immobilized enzyme still had high specific activity, 7.24 ± 2.07 U/mg protein. This result indicated that DIAION^®^CR11Co(II) might promote new interactions between the support and enzyme, which could produce hyperactivated forms of the enzyme to increase their expressed activities [16]. Based on the above results, in order to find a better immobilization method that has both high Chlase activity and couples with purification efficiency for industrial applications, recombinant BoCLH1 immobilized by the Cu(II)-loaded adsorbent was preferably used in the following experiments.

### 2.3.Characterization of the Immobilized Enzyme

Based on the results shown in Table S1, the purified recombinant BoCLH1 had higher Chlase activity for Chl a hydrolysis (36.8 ± 0.27) than other substrates. Therefore, we chose Chl a as a substrate for the Cu(II)-loaded immobilized BoCLH1 for determining biochemical properties such as pH and temperature influences. 

**Figure 3:**
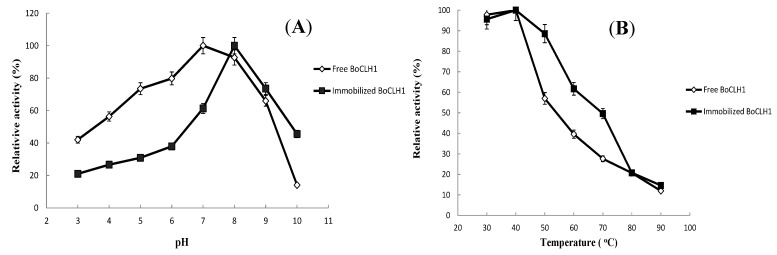
Effect of pH and temperature on the activity of free and immobilized BoCLH1. (**A**) Activity analysis of the free enzyme (◊) and the immobilized enzyme (■) were conducted at 40 °C and at pH 3 to 10 for 30 min. (**B**) Activity analysis of the free enzyme (◊) and the immobilized enzyme (■) were conducted at different temperatures (30–90 °C) for 30 min. A Cu(II)-loaded metal chelator was used for immobilization.

The effects of optimal pH and temperature on the activity of the free (crude cell lysate) and Cu(II)-loaded immobilized BoCLH1 were investigated. In Figure 3A, the immobilized enzyme exhibited an optimal reaction at pH 8.0. The immobilized enzyme activity declined significantly at pH below 7.0, but retained higher relative activity in a weak base environment. Compared to free enzyme, free enzyme exhibited an optimal reaction at pH 7.0, and retained higher relative activity at low pHs, but declined significantly at pH above 9.0. This result demonstrated that a free enzyme in an acidic environment has higher performance, but the immobilized enzyme can change its surface charge and conformation to maintain activity in a weak base environment [35,36]. 

As shown in Figure 3B, the optimal temperature of both the free and immobilized enzymes was 40 °C; as the temperature rose, activity for both the free and immobilized enzymes lessened. When the temperature exceeded 40 °C, the immobilized enzyme maintained a higher activity than that of the free enzyme at higher temperatures; for example, at 70 °C, a higher relative activity, 49.62%, is observed for the immobilized enzyme than that of 27.63% for the free enzyme. This result indicated that the immobilized enzyme has a high tolerance to temperature because the immobilized enzyme required a higher activation energy for reorganizing the structure appropriate to substrate binding [37]. On the other hand, the retention of activity at higher temperatures for immobilized enzyme was more stable, because the increased enzymatic activity with temperature can avoid enzyme conformational changes until the distortion of the enzyme structure is higher than the improvement in activity [21,38].

**Figure 4:**
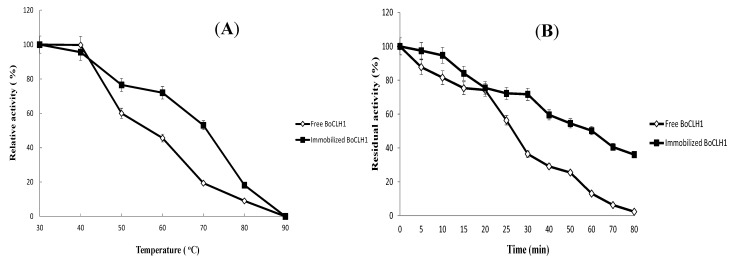
Thermostability of free and immobilized BoCLH1. (**A**) Free enzyme (◊) and the immobilized enzyme (■) were incubated at different temperatures (30–90 °C) for 30 min prior to activity analysis. (**B**) The thermal inactivation of the free enzyme (◊) and the immobilized enzyme (■) were incubated at 60 °C for various time intervals (0–80 min). A Cu(II)-loaded metal chelator was used for immobilization.

The effects of the thermal stability in the free and immobilized enzyme were studied. As shown in Figure 4A, the free enzyme had a higher activity at 40 °C than that of the immobilized enzyme, but, above 40 °C, the relative activity of the free enzyme decreased more sharply than that of the immobilized enzyme. At 60 °C, a relatively higher activity, 71.97%, was observed for the immobilized enzyme, compared with that of 45.59% for the free enzyme. The free enzyme operated at 60 °C but was easily inactivated with a temperature increase. We estimated that the half-life (*t*_1/2_) of the immobilized enzyme on DIAION^®^CR11Cu(II) BoCLH1 was 54.35 min at 60 °C (pH 7.0), whereas the free BoCLH1 was inactivated much faster under the same reactive condition (*t*_1/2_ 25.42 min) (Figure 4B). Therefore, compared with free BoCLH1, the immobilized BoCLH1 can enhance the half-life by approximately two-fold at 60 °C. This result explained how the immobilized enzyme can maintain enzyme activity at a high temperature for a long time due to immobilization-induced rigidification [21,23]. DIAION^®^CR11Cu(II) BoCLH1 immobilization could avoid distortion of the enzyme structure through coordination bonds between the chelated metal resin and the poly (His)-tagged protein.

### 2.4. Operational Stability

**Figure 5:**
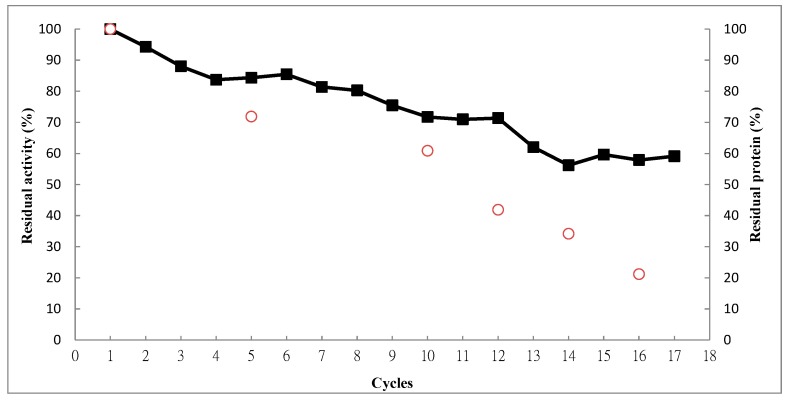
Reusability of the immobilized enzyme. Residual activity of the immobilized enzyme (■) and the amount of residual proteins (○) from the adsorbents were carried out at room temperature in a reaction buffer containing 100 μM Chl a for 30 min. A Cu(II)-loaded metal chelator was used for immobilization.

One of the advantages of the immobilized enzyme is that it can be reused to reduce costs. The operational stability of the immobilized recombinant BoCLH1 was monitored by determining the residual activity of the immobilized enzyme after each cycle at room temperature and pH 7 for 30 min. As shown in Figure 5, a residual activity of 59% was maintained after 17 cycles; however, after the 18th cycle, the residual activity decreased to 32%. The open circles in Figure 5 also indicate the protein capacity of the adsorbents; a reduction in the residual activity was associated with the absorbents’ decreasing protein load. Therefore, we re-loaded crude cell lysate with and without Cu^2+^ after 18 cycles, as shown in Figure S2; residual activities above 95% were measured, and the protein load of adsorbent on supports (the open circles) was also recovered in crude cell lysate with and without Cu^2+^, respectively. The gradual decline in residual activity could be ascribed to the desorption of recombinant BoCLH1 from the adsorbent, rather than as Cu^2+^ leached out from the support. Therefore, DIAION^®^CR11Cu(II) can be reused as a support to re-purify and re-immobilize these enzymes. The immobilized BoCLH1 had high operational stability after coordination bonds with the DIAION^®^CR11Cu(II)-loaded enzymes, and retained approximately 60% residual activity after 17 cycles. This is because the enzyme immobilized inside a porous solid can permit an “operational stabilization” of the enzyme, not affecting the structural stability of the enzyme [13]. This result clearly shows that immobilizing BoCLH1 coordination bonds with the DIAION^®^CR11Cu(II)-loaded enzymes is a successful method for decreasing biocatalyst costs in industrial application.

## 3. Experimental Section

### 3.1. Cloning and Plasmid Construction of BoCLH1 and Protein Expression 

We reported on *B. oleracea* Chlase genes named *BoCLH1* (GenBank accession number AF337544) [2]. The amplified *BoCLH1* fragment with the primer set (Forward: 5' GACGACGACAAGATGGGCATTCTCCGTTTTGTATTG 3'; Reverse: 5' GAGGAGAAGCCCGGAACCAGGTAACCAGAAGC 3') was cloned by polymerase chain reaction to pET-51b (+) Ek/LIC vector and carried N-terminal Strep-II-tag and optional carried C-terminal His-tag coding sequences. A recombinant plasmid was introduced into *E. coli* BL21 (DE3) to express the BoCLH1 protein. Transformants were selected with a Luria–Bertani medium (1% (w/v) Bactotryptone, 0.5% (w/v) Bacto yeast extract, 1% (w/v) NaCl, pH 7.0) with 50 μg/mL of ampicillin. The bacteria culture (OD_600_ = 0.6) was induced by adding 0.4 mM isopropyl β-d-1-thiogalactopyranoside (IPTG) at 16 °C for 16 h. After centrifugation at 4 °C for 10 min, the harvested cell pellet (from 1 L LB broth culture) was suspended in 10 mL of Tris buffer (0.5 M NaCl, 20 mM Tris-HCl, pH 7.9) and then frozen at −70 °C for 30 min. After thawing, the suspension was sonicated and then centrifuged (17,300 g for 20 min at 4 °C). The sonication conditions were 10 pulses at 30 s each with 30 s intervals set at 2.5 (approximately 137.5 W) (Sonicator XL-2020, Misonix Co., New York, NY, USA). The crude cell lysate was collected by centrifugation at 12,000 *g* for 20 min and filtered with a 0.22 μm filter.

### 3.2. Chlase Assay

The Chlase activity was analyzed following the method of Lee *et al.* [2]. The reaction mixture contained 10 μL of purified BoCLH1, 65 μL of a reaction buffer (100 mM sodium phosphate, pH 7.0, and 0.24% Triton X-100), and 7.5 μL of ethanol-dissolved Chl a from *Anacystis nidulans* algae (Sigma, St. Louis, MO, USA) at a final concentration of 100 μM. The reaction mixture was incubated in a shaking water bath at 40 °C from 0 to 30 min. The amount of product formed had a linear relationship with the reaction time of 30 min. Therefore, in the following assay, we carried out the reaction for 30 min to measure the initial velocity. The enzyme reaction was stopped by adding 1 mL of a stop-reaction buffer (ethanol/hexane/10 mM KOH = 4:6:1 (v/v)). The mixed solution was centrifuged at 20,000 *g* for 5 min for phase separation. The Chl a substrate was extracted in the hexane layer, and hydrolytic products (Chlide a) remained in the aqueous ethanol layer. The Chlide a in the aqueous ethanol phase was measured at 667 nm by using a spectrophotometer. The amount of product was estimated from millimolar extinction coefficients of 81.0 mM^−1^ cm^−1^ for the Chlide a. One unit (U) of enzyme activity was defined as 1 μmol of Chlide a from Chl a substrate per min at 40 °C.

### 3.3. Purification and Immobilization of the Enzyme

Ten grams of DIAION^®^CR11 (SUPELCO, Bellefonte, PA, USA) chelating resin were washed three times with 15 mL of Milli-Q water. The chelating resin was incubated with 40 mL of 200 mM FeCl_3_, Co(NO_3_)_2_, MnCl_2_, ZnSO_4_, NiSO_4_, or CuSO_4_, depending on the type of metal ions desired, at room temperature for 3 h. The chelating resin charged with metal ions was washed twice with 40 mL of a 50 mM phosphate buffer (pH 7.0) and used for enzyme purification and immobilization. Next, we took 0.1 g dry weight of each chelating resin charged with a different metal ion and this was poured into 1 mL of crude cell lysate (containing overexpression the recombinant BoCLH1 protein) for mixing 3 h at room temperature. After 3 h incubation, protein-loaded adsorbents were separated using a filter, and the spent cell lysate was monitored using 12% SDS-PAGE and western blotting. The purified BoCLH1 protein (38.63 kDa) was set as a positive control by using a StepTrap^TM^ HP affinity chromatography system (GE Healthcare, Inc., Piscataway, NJ, USA)following the manufacturer’s instructions. A monoclonal anti-His tag antibody (BioRad, MCA1396) was used for deterring the BoCLH1.

To analyze the protein-binding capacity of the chelating resin, the immobilized enzyme was eluted with 1 mL 50 mM phosphate buffer containing 500 mM imidazole (pH 7). The eluted fractions were analyzed using the Bradford protein assay.

### 3.4. Biochemical Analyses of Crude and Immobilized BoCLH1

To assess their optimal pH levels, the crude and immobilized enzymes were each investigated in a 50 mM sodium acetate buffer with a pH range from 3 to 5 and in a Good’s buffer (50 mM each of Bicine, CAPS, and Bis-Tris propane) with a pH range from 6 to 10. The reaction mixture contained 20 μL of crude BoCLH1 or 0.1 g of immobilized BoCLH1, 195 μL of a reaction buffer (pH 3–10), and 22.5 μL of ethanol-dissolved Chl a (100 μM) for 30 min at 40 °C. 

To examine the optimal temperature, the crude and immobilized enzymes were each incubated with Chl a in 100 mM sodium phosphate, pH 7.0 with 0.24% Triton X-100, at a temperature range from 30 to 90 °C for 30 min to measure its activity dependence on the temperature. To analyze thermal stability, the crude and immobilized enzymes were each incubated for 10 min at a temperature range from 30 to 90 °C and subsequently cooled down prior to activity analysis. The relative activity was measured at 667 nm using a spectrophotometer at 40 °C for 30 min. 

To test the half-life times, the crude and immobilized enzymes were each incubated in the presence of 100 mM sodium phosphate, pH 7.0 with 0.24% Triton X-100, at 60 °C for 0–80 min, and then the residual activity was measured at different incubation times. The initial activity levels were assessed under conditions of 40 °C for 30 min and taken to represent 100%. The half-life times were estimated according to Zhou *et al.* [39] and Lam *et al.* [40]. 

### 3.5. Operational Stability

The operational stability of the immobilized enzyme was monitored by determining the residual activity of the immobilized enzyme after each cycle. The reaction was performed in 100 mM sodium phosphate with 0.24% Triton X-100 at room temperature for 30 min. After each cycle, the immobilized enzyme was recovered by filtration. To analyze the residual protein, the immobilized enzyme was eluted with 1 mL 50 mM phosphate buffer containing 500 mM imidazole (pH 7). The eluted fractions were analyzed using the Bradford protein assay.

## 4. Conclusions

In summary, we used a polystyrene divinylbenzene-based metal chelator such as DIAION^®^CR11 to purify and immobilize recombinant poly(His)-tagged recombinant BoCLH1. Cu(II) exhibited the highest capacity (9.2 ± 0.43 mg/g gel) and purification efficiency, therefore DIAION^®^CR11Cu(II) was the preferred enzyme immobilization for this study. There were significant changes in optimal pH and temperature between the free and immobilized enzymes. The immobilized enzyme exhibited slightly higher activity in a weak base environment (pH 8) and at 40 °C. The effects of the thermal stability indicated that the immobilized BoCLH1 could enhance residual activities by approximately two-fold at 60 °C. The operational stability of DIAION^®^CR11Cu(II)-recombinant BoCLH1 maintained approximately 60% residual activity after 17 cycles. Re-loading the crude cell lysate, with or without Cu^2+^, can recover the residual activities of immobilized BoCLH1. Therefore, based on the above results, DIAION^®^CR11Cu(II)-recombinant BoCLH1 is a successful immobilized enzyme that can be reused to reduce costs in an industrial application.

## Supplementary Materials

Supplementary materials can be accessed at: http://www.mdpi.com/1420-3049/20/03/3744/s1.
