# Supplementary material for: Purification and Immobilization of the Recombinant Brassica oleracea Chlorophyllase 1 (BoCLH1) on DIAION®CR11 as Potential Biocatalyst for the Production of Chlorophyllide and Phytol
Source: Molecules. 2015 Feb 24;20(3):3744–57. doi: 10.3390/molecules20033744 (PMC6272265; doi:10.3390/molecules20033744)
Supplement: Supplementary file 1 [file molecules-20-03744-s001.pdf]

Supplementary Materials

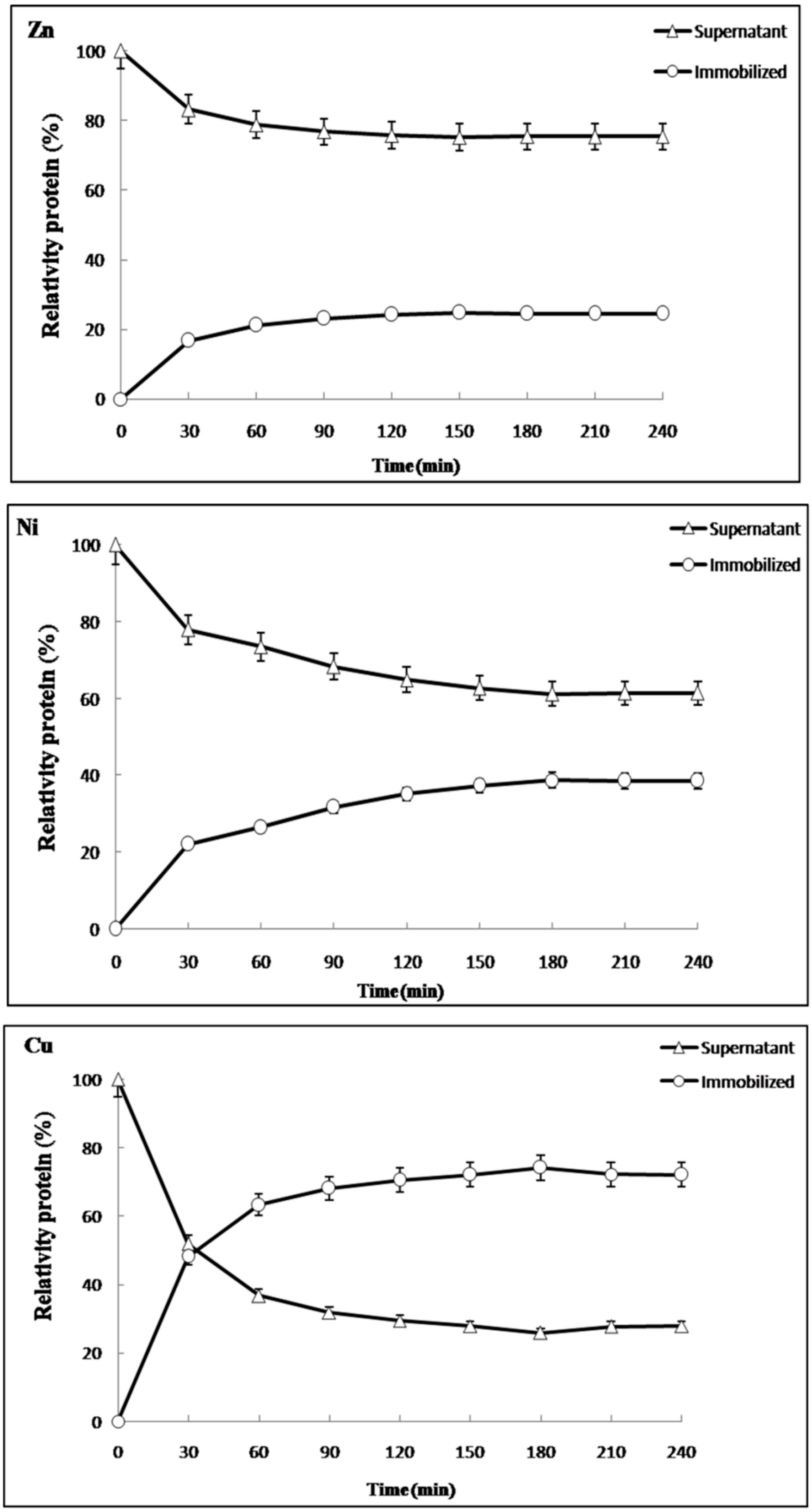

Figure S1. *Cont*.

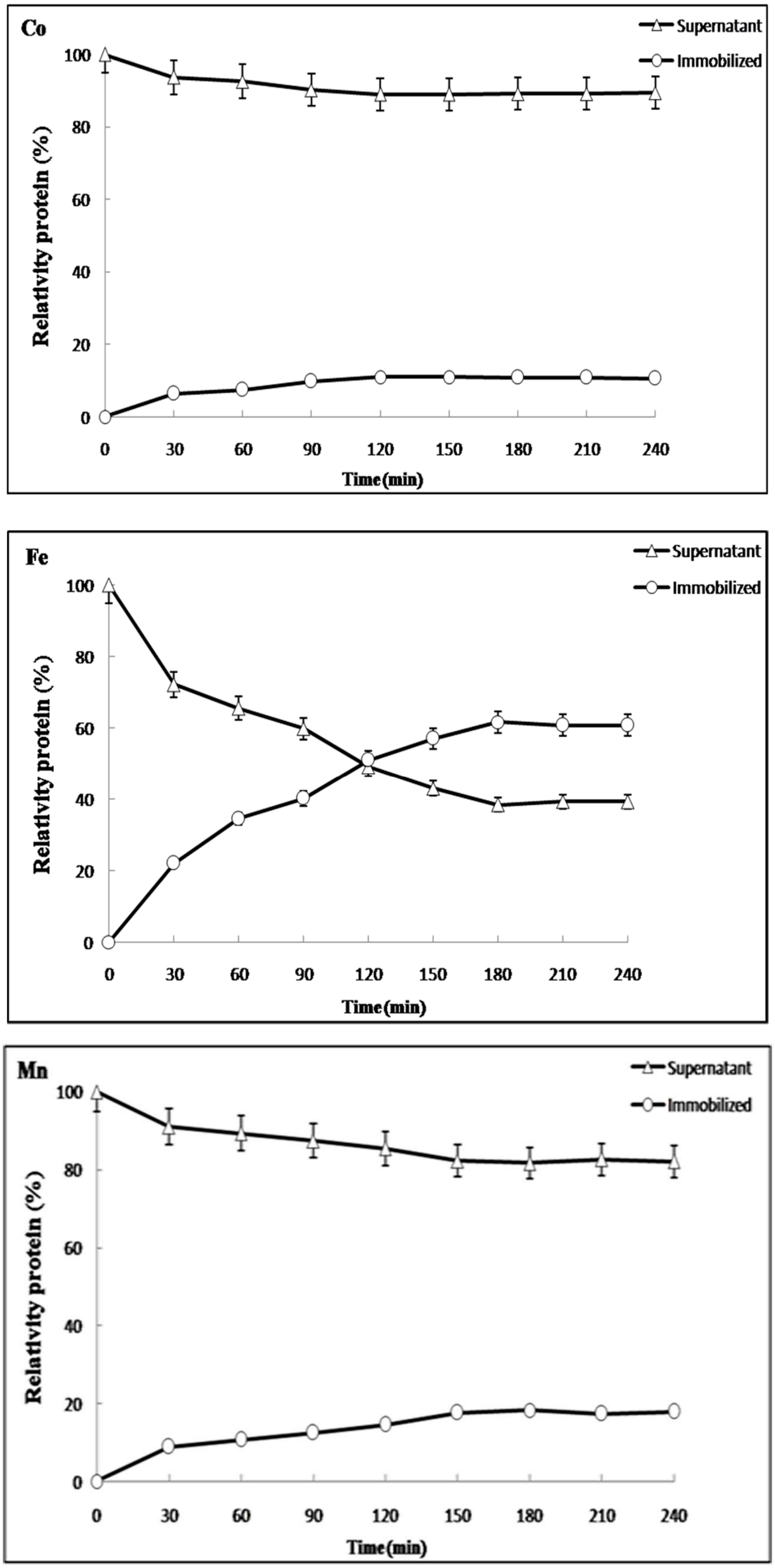

Figure S1. Immobilization curves of the DIAION®CR11 chelated six metal ions to capture poly (His)-tagged of recombinant BoCLH1 in 1 mL crude cell lysate. Relativity protein of the immobilized (○) and the supernatant (Δ) were analyzed using the Bradford protein assay.

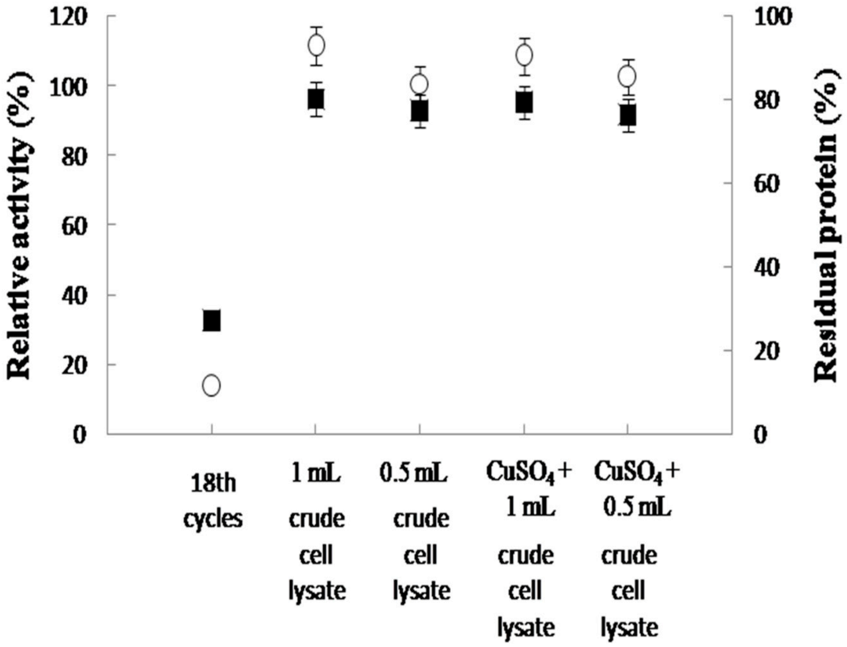

Figure S2. Reloading crude cell lysate in the DIAION®CR11 with and without adding $CuSO_4$ after 18 cycles. Relative activity of the immobilized enzyme (■) and the amount of immobilized proteins (○) from the adsorbents were carried out at room temperature in a reaction buffer containing 100 µM Chl a for 30 min. The first cycle of the specific activity and adsorbents as 100%.

Table S1. Substrate specificity of purified BoCLH1 in the hydrolysis of chlorophylls, *p*-nitrophenyl (*p*-NP) esters and amino acid derivatives.

**Substrate**	**BoCLH1** [a] **(units/mg)**
Chlorophyll [b]	
Chlorophyll a	36.8 ± 0.27 (100%)
Chlorophyll b	20.8 ± 0.49 (56.49%)
Bacteriochlorophyll a	0.90 ± 0.06 (2.34%)
p-Nitrophenyl (p-NP) esters [c]	
p-NP Acetate (2C)	0.46 ± 0.002 (100%)
p-NP Butyrate (4C)	0.17 ± 0.003 (36.15%)
p-NP Caproate (6C)	0.28 ± 0.032 (60.25%)
p-NP Caprylate (8C)	0.21 ± 0.003 (45.21%)
p-NP Caprate (10C)	0.15 ± 0.004 (31.54%)
p-NP Laurate (12C)	0.10 ± 0.008 (21.26%)
p-NP Myristate (14C)	0.07 ± 0.004 (16.21%)
p-NP Palmitate (16C)	0.08 ± 0.004 (18.07%)
p-NP Stearate (18C)	0.04 ± 0.030 (7.64%)
Amino acid derivatives [d]	
L-NBPNPE	0.07 ± 0.004 (56.93%)
D-NBPNPE	0.12 ± 0.001 (100%)
L-NBTNPE	0.08 ± 0.004 (63.19%)

[a] The purified BoCLH1 was eluted with a nickel-elution buffer and StepTrap™ HP affinity chromatography system. [b] One unit (U) of enzyme activity was defined as the amount of enzyme that hydrolyzed 1 μmol of Chls per min at 40 °C. [c] One unit of esterase activity is the amount of enzyme that hydrolyses 1 μmol of *p*-NP ester per min at 40 °C and pH 8.0. [d] Substrates of amino acid-derived esters: *N*-carbobenzoxy-L-phenylalanine *p*-nitrophenyl ester (L-NBPNPE), *N*-carbobenzoxy-D-phenylalanine *p*-nitrophenyl ester (D-NBPNPE) or *N*-carbobenzoxy-L-tyrosine *p*-nitrophenyl ester (L-NBTNPE). One unit was defined as catalyzing the hydrolysis of 1 μmol substrate to produce 1 μmol of *p*-nitrophenol per min. Values are means ± S.D. from three independent experiments.

Substrate Specificity

Chlase Assay

The Chlase activity was analyzed following the method of Lee *et al.* [1], with minor modification. The reaction mixture contained 10 μL of purified BoCLH1, 65 μL of a reaction buffer (100 mM sodium phosphate, pH 7.0, and 0.24% Triton X-100), and 7.5 μL of ethanol-dissolved Chl a from *Anacystis nidulans* algae, Chl b from *spinach*, and BChl a from *Rhodopseudomonas sphaeroides* (Sigma, St. Louis, MO, USA) at a final concentration of 100 μM. The reaction mixture was incubated in a shaking water bath at 40 °C from 0 to 30 min. The amount of product formed had a linear relationship with a reaction time of 30 min. Therefore, in the following assay, we carried out the reaction for 30 min to measure the initial velocity. The enzyme reaction was stopped by adding 1 mL of a stop-reaction

buffer (ethanol/hexane/10 mM KOH = 4:6:1 (v/v)). The mixed solution was centrifuged at 20,000 × *g* for 5 min for phase separation. The Chl substrates were extracted in the hexane layer, and hydrolytic products (Chlide a, Chlide b, and BChlide a) remained in the aqueous ethanol layer. The Chlide a, Chlide b, and BChlide a in the aqueous ethanol phase were measured at 667, 646, and 771 nm, respectively, using a spectrophotometer. The amount of each product was estimated from millimolar extinction coefficients of 81.0, 47.6, and 76.0 mM^{-1} cm^{-1} for Chlide a, Chlide b, and BChlide a, respectively. One unit (U) of enzyme activity was defined as 1 μmol of Chlide a, Chlide b, and BChlide a from different Chl substrates per min at 40 °C.

Esterase Activity Assay

The 10 μL of purified BoCHL1 solution was added into 200 μL of reaction solution (50 mM sodium phosphate buffer, pH 7.0, and 2.0% Triton X-100) containing 2.5 mM *p*-NP ester of acetate (2C), butyrate (4C), caproate (6C), caprylate (8C), caprate (C10), laurate (C12), myristate (C14), palmitate (C16), or stearate (C18) as substrates. The reactions were incubated at 40 °C for 1 h. The *p*-nitrophenol from substrate hydrolysis was measured at 405 nm using a spectrophotometer. The molar extinction coefficient of the *p*-nitrophenol in this buffer system was 1,725 M^{-1} cm^{-1}.

Amino Acid-Derivative Substrate Assay

Stock solutions (2.0 mM) of the amino acid-derived substrates, including the *N*-carbobenzoxy-L-phenylalanine *p*-nitrophyl ester (L-NBPNPE), *N*-carbobenzoxy-D-phenylalanine *p*-NP ester (D-NBPNPE), or *N*-carbobenzoxy-L-tyrosine *p*-NP ester (L-NBTNPE), were prepared in 1 mL of dioxane. The 10 μL of purified BoCHL1 was incubated with 0.01 mM amino acid-derived substrates in 0.2 mL of a 100 mM sodium phosphate buffer (pH 7.0) at 40 °C for 1 h. The *p*-nitrophenol was estimated at 400 nm using a spectrophotometer. One unit was defined as catalyzing the hydrolysis of 1 μmol substrate to produce 1 μmol of *p*-nitrophenol per min. The molar extinction coefficient was 3,454 M^{-1} cm^{-1} under these conditions.

Reloading Crude Cell Lysate in the Support with and without Adding Cu^{2+}

After 18 cycles of consecutive reaction, the remaining supports were separated using a filter and then 400 μL of reloaded 200 mM $CuSO_4$ was re-incubated for 3 h at room temperature or 1 mL or 0.5 mL of crude cell lysate was mixed for 3 h at room temperature. The re-incubated support was washed twice with 40 mL of a 50 mM phosphate buffer (pH 7.0) and separated using a filter, then added to 1 mL or 0.5 mL of crude cell lysate for 3 h of mixing at room temperature. Next, protein-loaded adsorbents were analyzed for the Chlase activity assay and the protein-binding capacity of the supports was measured.

Reference

1. Lee, G.C.; Chepyshko, H.; Chen, H.H.; Chu, C.C.; Chou, Y.F.; Akoh, C.C.; Shaw, J.F. Genes and biochemical characterization of three novel chlorophyllase isozymes from *Brassica oleracea*. *J. Agric. Food Chem.* **2010**, *58*, 8651–8657.
